# Coupled Thermogravimetric Analysis-Potentiometric Titration for Complex Analysis of Poly(vinyl chloride) Thermal Stability

**DOI:** 10.1021/acsmeasuresciau.4c00090

**Published:** 2025-01-03

**Authors:** Jonáš Uřičář, Anežka Chodounská, Václava Benešová, Jiří Brožek, Radka Kalousková

**Affiliations:** † Department of Polymers, University of Chemistry and Technology Prague, Technická 5, 166 28 Prague 6, Czech Republic

**Keywords:** poly(vinyl chloride), dehydrochlorination, thermal stability, thermogravimetric analysis, potentiometric titration

## Abstract

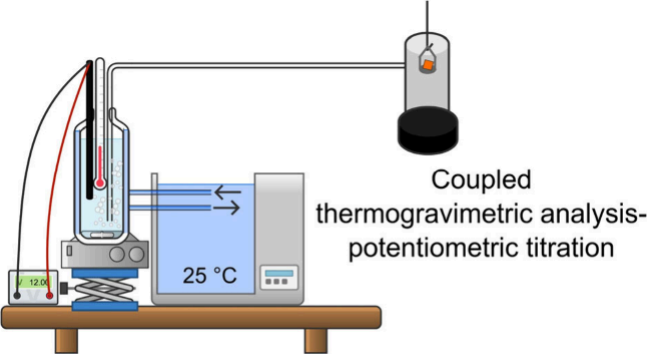

Degradation of poly(vinyl chloride) is a widely discussed topic, and its thermal stability is one of its most important properties. This work uses coupled thermogravimetric analysis-potentiometric titration for simultaneous analysis of sample weight loss and quantification of released hydrogen chloride. The thermal stability point of highly plasticized samples cannot be determined from thermogravimetric measurement alone, as the weight loss derivative change is not clearly visible. This problem is solved by the presented method, which was applied to both unplasticized and plasticized samples. The obtained data can be used to identify the thermal stability point and separate the mass loss caused by the released hydrochloric acid and by other compounds. Such data can be used in the future for determination of more precise parameters for degradation kinetics models.

The thermal stability (TS) of poly(vinyl chloride) (PVC) in isothermal mode is one of its most important properties. Several studies addressed this issue,^1−5^ and data can be utilized to predict the material performance.^6^ TS is characterized by the time for which no or almost no hydrogen chloride is released from the thermally stressed PVC compound (for instance at 180 °C).^7^ The TS value often is accompanied by a dehydrochlorination (DHC) curve measured by accurate and reproducible continuous potentiometric titration.^8^ The DHC mechanism has already been thoroughly described in the literature.^9,10^

Several kinetics models can be used for the description of DHC.^11,12^ Cruz et al. calculated kinetic parameters for DHC utilizing dynamic thermogravimetric measurement of pure PVC without any additives.^13^ Oh et al. performed a similar experiment utilizing thermogravimetric analysis (TGA) of waste PVC insulation, but also used a static quartz tube reactor for the DHC and caught the gaseous degradation products into PTFE bags. Such gaseous products were afterward analyzed via gas chromatography–mass spectrometry (GC-MS) to determine their composition. The authors showed that the amount of CO_2_ and organic compounds in the studied gas can be higher in comparison with HCl.^14^ Torres et al. performed TGA of samples containing both PVC and varying HCl scavengers and estimated the average mass at a given temperature from masses of PVC and hydrochloride removal at the same temperature.^15^ Zhao et al. described different approaches to study dehydrochlorination. They used hydrothermal treatment with capture of emerging gas followed by combustion-ion titration to determine the chlorine content.^16^

The authors of this work propose that using only TGA for determination of kinetics parameters of DHC and the TS is not precise enough, as it might be influenced by weight loss of other compounds. Li et al. used coupled TGA-MS to characterize the weight loss and HCl release behavior during thermal degradation.^17^ This method did not directly lead to the quantification of the released HCl. Also, coupling TGA with advanced analytical instruments, such as a mass spectrometer, is not recommended by the authors of this work, as it is known that HCl is corrosive^18^ and the authors encountered damage to the instrument in the preliminary experiments.

Therefore, herein, we report isothermal thermogravimetric analysis coupled with potentiometric titration (TGA-PT) for complex analysis of isothermal dehydrochlorination, including weight loss dependent on degradation time, quantification of released HCl, and the TS of PVC.

The experimental setup is schematically depicted in Figure 1. The gas exhaust of the furnace of the Discovery TGA550 Auto Advanced (TA Instruments, USA) was connected with AgNO_3_ solution by PTFE tube with PE tip, which was used to obtain smaller bubbles size. The AgNO_3_ solution (Safina Czech Republic, concentration 14.61 μmol Ag^+^ L^–1^ in deionized water) was tempered in a double-wall glass bottle with an external circulation thermostat. The solution was magnetically mixed during experiments to ensure a homogeneous concentration and quantitative reaction. Measurement electrodes (reference calomel and silver) and a digital thermometer were used to record the potential and exact temperature, utilizing a potentiometer (multimeter Hanna Instruments 931).

**Figure 1:**
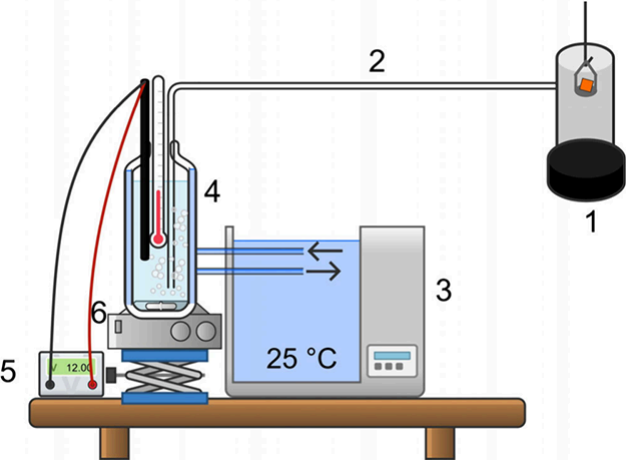
TGA-PT setup: (1) TGA furnace with gas outlet; (2) PTFE gas transfer tube; (3) circulation thermostat;(4) externally tempered double-wall glass bottle; (5) advanced potentiometer connected to the electrodes and thermometer; (6) magnetic stirrer.

Measurements of two samples are presented in this work. The materials used in preparation of samples were PVC Neralit 682 (K-values of 67–69, Spolana Neratovice, CZ), heat stabilizer Stabilox GTU 1233/1 (Reagens, SRN), plasticizer Kodaflex DOTP (Eastman Kodak Company, USA), and lubricant Bralen (Slovnaft a.s., SK). PVC mixtures in the form of foils were prepared on a Collin W 100T two-roll mill with the following: rolling temperature, 170 °C; mixture preparation, time 7 min. **Sample 1** contains 3 wt parts of Stabilox GTU per 100 wt parts of PVC and 1 wt part of Bralen per 100 wt parts of PVC. **Sample 2** contains 3 wt parts of Stabilox GTU per 100 wt parts of PVC, 1 wt part of Bralen per 100 wt parts of PVC and 40 wt parts of DOTP per 100 wt parts of PVC.

Both were measured by the presented coupled method; TGA temperature setting was 40 °C/min ramp from room temperature up to 180 °C followed by isothermal mode at this temperature, air sample purge flow was 60 mL·min^–1^, and balance flow was 40 mL·min^–1^. Sample weight was measured by TGA, and the potential of the solution was measured via the used multimeter.

The measured potential was used to calculate the activity of silver ions using the modified Nerst equation (eq 1) at a given degradation time:
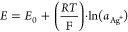
1where *E* is the measured potential [V], *E*_0_ is the standard potential [V], R is the universal gas constant [J·mol^–1^·K^–1^], *T* is the temperature [K], F is the Faraday constant [C·mol^–1^], and *a*_*Ag*_^*+*^ is the activity of the silver ions (for our calculation we used concentration [mol·L^–1^]).

It is well-known that the reaction between AgNO_3_ and HCl has the stoichiometry 1:1; thus, the concentration of Ag^+^ ions at a given degradation time was used to calculate the absolute mass of HCl that entered the solution. The mass of HCl at a given time was afterward subtracted from the initial sample mass; the obtained curve and the TGA curve were plotted into the same graph.

Figure 2 shows the results for sample 1 (unplasticized). There are clear differences between weight loss caused by removed HCl (red) and total weight loss (blue). DHC data were linearly extrapolated from regions 480–1020 s and 1500–1980 s, respectively (dashed), to obtain their interception, the TS, which was found around 1350 s. Measurement of this sample by continual potentiometric titration led to comparable results; the TS was lower by about 4 min, which is the time to reach 180 °C in the TGA machine.

**Figure 2:**
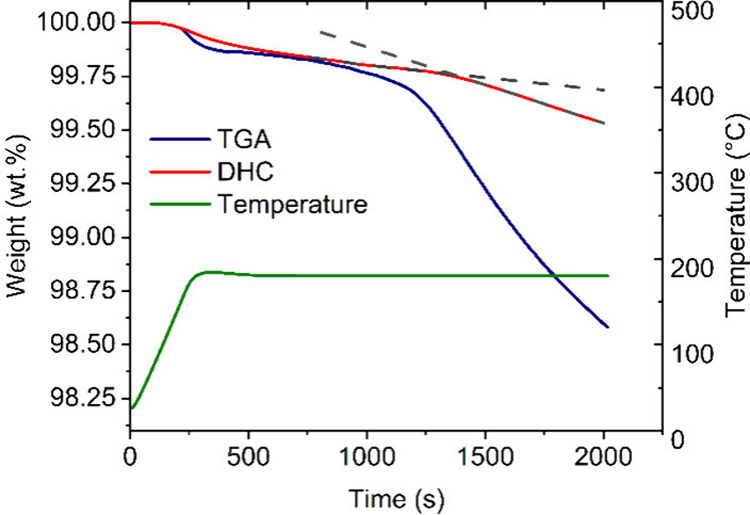
TGA:DHC plot of sample 1 (unplasticized).

In our preliminary experiments, we encountered a problem that the weight change derivative at certain sample TSs is not visible in the TGA curve (or in its derivative) when measuring highly plasticized samples. This problem was an inspiration to create the presented coupled method. Such a problem is shown in Figure 3, which depicts the obtained results from sample 2 (plasticized). The overall weight loss is significantly higher for the plasticized sample in comparison with that of the unplasticized one.

**Figure 3:**
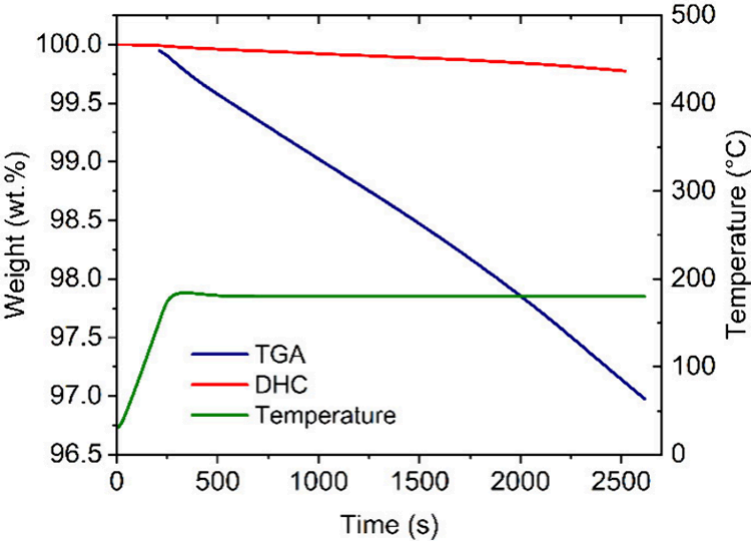
TGA:DHC plot of sample 2 (plasticized).

For better visibility, Figure 4 divides TGA (blue) and DHC (red) data into separated *y*-axis. It is clear that TS cannot be determined by TGA alone for this sample. Therefore, TS was identified from interception of the linear extrapolation of DHC data (dash curves) from regions 1020–1500 s and 2040–2520 s, respectively, around 1800 s. Measurement of this sample by continual potentiometric titration led to comparable results; the TS was lower by about 4 min, which is the time to reach the 180 °C in the TGA machine. The advantage of TGA-PT is that we know the temperature profile, even before reaching the equilibrated degradation temperature.

**Figure 4:**
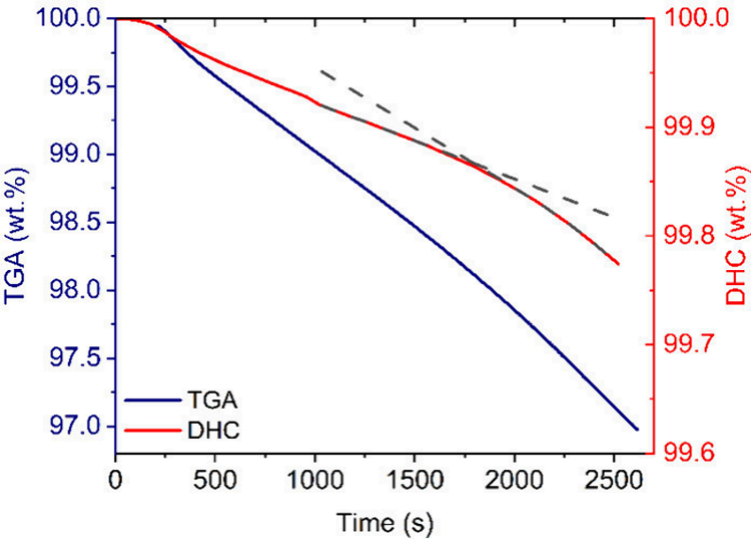
TGA:DHC plot of sample 2 (plasticized) with zoom.

This work presented a coupled thermogravimetric analysis-potentiometric titration method that overcomes the research gap in the evaluation of thermal stability of the plasticized PVC samples while measuring the sample weight, as the thermal stability point cannot be determined from the thermogravimetric analysis alone for such materials. During this analysis, the differences between the overall weight loss and weight loss caused by the quantified hydrogen chloride were shown, and it was clearly visible that the weight loss caused by other compounds is significant. The separation of these weight losses can be used in the future for determination of more precise parameters for degradation kinetics models in both isothermal and dynamic modes.

## Data Availability

Dataset available at https://doi.org/10.5281/zenodo.14587622.
